# The prevalence of workplace violence toward psychiatric nurses in Saudi Arabia and its effect on their quality of life

**DOI:** 10.3389/fpsyt.2025.1524845

**Published:** 2025-03-20

**Authors:** Sae’d M. Abu El-Kass, Osama Mahmoed Ellayan, Anas Muhmmad Turkman, Hind Mushabab Al Mansour, Musherh Alraied Alrowily, Khairyah Abdullah Alsobhan, Bushra Alraydh Alruwaili, Norah Saud Alqahtani, Hana Alraydh Alruwaili, Abdel Hamid El Bilbeisi

**Affiliations:** ^1^ Department of Nursing, Faculty of Nursing and Health Sciences, University College of Applied Sciences, Gaza, Palestine; ^2^ Department of Nursing, Faculty of Medical Sciences, Al-Aqsa University, Gaza, Palestine; ^3^ Department of Nursing, Faculty of Nursing and Medical Sciences, Gaza University, Gaza, Palestine; ^4^ Department of Nursing, Eradah Complex for Mental Health, Arar, Saudi Arabia; ^5^ Department of Nursing, Ministry of Health, Aseer, Saudi Arabia; ^6^ Department of Obstetrics and Gynecology, Maternity, Women and Children’s Hospital, Ministry of Health, Al-Jouf, Saudi Arabia; ^7^ Department of Nursing, Aseer Health Cluster, Aseer, Saudi Arabia; ^8^ Maternity and Children Hospital-Arar, Ministry of Health, Arar, Saudi Arabia; ^9^ King Abdulaziz Specialist Hospital, Ministry of Health, Al-Jouf, Saudi Arabia; ^10^ Department of Clinical Nutrition, Faculty of Pharmacy, Al Azhar University of Gaza, Gaza, Palestine

**Keywords:** nurses, prevalence, quality of life, violence, workplace

## Abstract

**Background:**

Workplace violence is a significant cause of work-related stress in nursing, affecting job performance and satisfaction and increasing burnout risk. This study aims to evaluate the prevalence of verbal and physical violence against psychiatric nurses and its impact on their quality of life.

**Methods:**

This cross-sectional study was conducted from April to October 2024. A sample of 171 nurses was selected using a convenience sampling method. The study was carried out at Eradah Complex for Mental Health in Arar City, Eradah Hospital for Mental Health in Al Jouf City, and Mental Health Hospital in Al Qurayyat City. The authors evaluated workplace violence and quality of life among nurses using a questionnaire-based interview and a 36-item quality of life assessment tool. Statistical analysis was performed using SPSS version 25.

**Results:**

The participants’ mean age was 34.85 ± 4.74 years, 67.8% were male, and 52.7% had over 10 years of experience. The overall prevalence of workplace violence was found to be 100%, with 62% exposed to verbal violence and 38% to physical violence. Regarding quality of life, 66% had an average quality of life, 12.9% had a good quality of life, and 21.1% had a poor quality of life. Statistically significant associations were found between the type of violence and quality of life (P-value < 0.05).

**Conclusion:**

The study found significant levels of verbal and physical workplace violence among psychiatric nurses, with a significant correlation between violence and their quality of life. These results highlight the need for targeted interventions, including training programs, improved workplace safety policies, and continuous monitoring to support nurse well-being and job satisfaction.

## Introduction

Violence involves the intentional use of physical force, power, threats, or actual harm directed at oneself, others, a group, or a community, with the potential to cause damage, death, psychological harm, developmental issues, or deprivation. It can manifest in various forms, including verbal abuse, bullying, harassment, and physical acts such as kicking, pushing, or biting. Workplaces can also be environments where violence occurs (1).

Workplace violence against nurses in healthcare settings is common worldwide, negatively affecting both nurses and organizations and potentially lowering care standards. Psychiatric nurses, especially in acute and long-term care facilities, face significant risks due to frequent exposure to violent patient behavior, threatening their emotional, physical, and psychological wellbeing (2).

The World Health Organization (WHO) defines quality of life as an individual’s evaluation of their life circumstances in relation to the cultural and value system they are part of, along with their goals, expectations, peers, and concerns. It is a comprehensive concept that is deeply shaped by a person’s physical and mental health, psychological state, beliefs, social connections, and relationship with intangible aspects of their environment (3).

Itzhaki et al. (4) found that workplace violence in nursing affects job performance, recruitment, retention, and overall quality of life for professionals. Similarly, Choi and Lee (2017) (5) reported that nurses in psychiatric wards experienced the highest incidence of all three forms of violence: verbal abuse, physical threats, and actual violence. These nurses also had the highest levels of secondary trauma and a lower quality of life compared to those who had not experienced all three types of violence.

Workplace violence is a major contributor to work-related stress in nursing, negatively impacting job performance, job satisfaction, and increasing the risk of burnout. Psychiatric nurses, in particular, face higher levels of stress compared to nurses in general hospitals, making them more prone to experiencing work stress-related symptoms (4).

Additionally, workplace violence can negatively impact the standard of service in healthcare settings by affecting nurses’ physical and mental wellbeing, leading to decreased job performance, lower productivity, and increased absenteeism. Nurses who experience violence may also have lower job satisfaction, higher burnout rates, and a diminished ability to provide compassionate care. This can result in compromised patient care, reduced quality of services, and potentially higher turnover rates, which disrupts the continuity of care and overall organizational effectiveness. In the long term, these effects can contribute to a decline in the overall quality of healthcare services provided to patients (4, 5).

To the best of our knowledge, this is one of the first studies to examine the prevalence of workplace violence among psychiatric nurses in Saudi Arabia. Although some previous studies have explored workplace violence in healthcare settings, particularly in general hospitals, there is limited research specifically focused on psychiatric nurses in Saudi Arabia. Given the unique challenges faced by psychiatric nurses, including frequent exposure to aggressive behavior, it is crucial to understand how verbal and physical violence affects their wellbeing. This study aims to assess the prevalence of verbal and physical workplace violence against psychiatric nurses and its impact on their quality of life. By addressing this gap in research, the study will provide valuable insights that can inform targeted interventions, improve workplace safety, and support the mental and physical health of psychiatric nurses in Saudi Arabia, ultimately enhancing the quality of care provided to patients.

## Materials and methods

### Study design and period

This cross-sectional study was conducted between April and October 2024 to assess the prevalence of verbal and physical workplace violence against psychiatric nurses and its impact on their quality of life in Saudi Arabia.

### Study setting

This study was carried out in Saudi Arabia at the Eradah Complex for Mental Health in Arar City, Eradah Hospital for Mental Health in Al Jouf City, and the Mental Health Hospital in Al Qurayyat City.

### Study participants and sampling technique

A total of 171 nurses were selected using a convenience sampling method for this study. All available psychiatric nurses, aged 18 to 60 years, who had worked for at least 6 months in the specified settings, were included, regardless of sex. The study participants who met the inclusion criteria were distributed as follows: 55 nurses from Eradah Complex for Mental Health, 68 nurses from Eradah Hospital for Mental Health, and 48 nurses from the Mental Health Hospital.

### Eligibility criteria

The study included all available psychiatric nurses, regardless of sex, aged 18 to 60 years, who had worked for at least 6 months in the aforementioned settings. Exclusion criteria consisted of volunteers, other healthcare providers, and individuals with less than 6 months of work experience in the specified settings. Additionally, nurses on long-term medical leave, those with a history of severe mental health conditions, and those not actively involved in direct patient care were also excluded from the study.

### Data collection

In this study, all participating psychiatric nurses were evaluated using an interview-based questionnaire. Additionally, data on their quality of life was collected using a 36-item quality of life assessment tool.

### Interview-based questionnaire

This questionnaire was developed by the investigator after reviewing relevant literature and was written in simple Arabic to collect data on the following: 1) the sociodemographic characteristics of the nurses (six questions) and 2) the prevalence and characteristics of workplace violence among nurses (eight questions). The tool, adapted from previous studies (6, 7), was designed to assess both verbal and physical violence, including factors such as frequency, type of violence, perpetrators, work shifts, and the nurses’ responses to violence. The study tool was adapted from previous studies by revising certain questions and adding new items to better reflect the sociodemographic and contextual factors relevant to this study.

### Assessment of quality of life

In this study, the Medical Outcomes Study’s 36-Item Short-Form Health Survey (SF-36) was used to assess the impact of workplace violence on the quality of life of the nurses. This tool, adapted from previous studies (8–10), evaluates eight domains of quality of life with a five-point Likert scale.

### Scoring system

The quality of life assessment tool consists of 36 items, divided into eight domains, with a total score of 148. The scores are classified as follows: poor quality of life (< 60%), corresponding to a total score of less than 88.8; average quality of life (60% to < 75%), corresponding to a total score between 88.8 and 110.9; and good quality of life (≥ 75%), corresponding to a total score of 111 or higher (8–10).

### Content validity and reliability

Face and content validity were evaluated by a panel of three experts (two assistant professors and one lecturer) from the Faculty of Nursing at Damietta University and Beni Suef University. The experts reviewed the tools for clarity, relevance, comprehensiveness, simplicity, and applicability. After making minor modifications, the final versions were developed. Additionally, the reliability of the tools was assessed using Cronbach’s alpha coefficients: 0.825 for the prevalence of workplace violence and its characteristics among nurses, and 0.746 for the Medical Outcomes Study’s 36-Item Quality of Life Health Survey Short-Form.

Furthermore, to reduce the likelihood of over- or under-reporting variables, we employed a number of strategies. Initially, we utilized validated and reliable measurement tools to maintain consistency in data collection. We also provided participants with clear instructions to minimize any potential misunderstandings of the questions. Through these measures, we aimed to ensure that our findings were both precise and unbiased.

### Pilot study

A pilot study was conducted with 10% of the sample size (17 nurses) to assess the tools’ applicability, clarity, and effectiveness. No modifications were made based on the pilot study results, and the nurses who participated were included in the main study sample without any changes.

### Data analysis

Statistical analysis was conducted using SPSS version 25. The normality of the data was evaluated using both the Kolmogorov–Smirnov and Shapiro–Wilk tests, with p-values greater than 0.05 indicating normal distribution. Data are presented as means ± SD for continuous variables and as percentages for categorical variables. Independent sample t-tests were used to compare means, while the Chi-square test was employed to examine differences in the prevalence of categorical variables. A p-value of less than 0.05 was considered statistically significant.

## Results

**Table 1** shows the sociodemographic characteristics of the study participants by sex. The results revealed that 39.8% of the nurses were from Eradah Hospital for Mental Health in Al Jouf City, 32.2% were from Eradah Complex for Mental Health in Arar City, and 28.0% were from Mental Health Hospital in Al Qurayyat City. A large percentage, 69.6%, of the nurses were in the 31–40 years age group, with a mean age of 34.85 ± 4.74 years. Of the participants, 67.8% were male, 83.0% were married, 60.2% held a nursing diploma, 71.9% worked as bedside nurses, and 52.7% had more than 10 years of experience. Regarding marital status, 71.8% of the male nurses were married, compared to 28.2% of the female nurses.

**Table 1:** Association between the sociodemographic characteristics of study participants and sex (N=171).

Variable	Total, n (%) 171 (100)	Male, n (%) 116 (67.8)	Female, n (%) 55 (32.2)	p-value
Hospital name				
Eradah Complex for Mental Health	55 (32.2)	31 (56.4)	24 (43.6)	0.078
Eradah Hospital for Mental Health	68 (39.8)	51 (75.0)	17 (25.0)	
Mental Health Hospital	48 (28.0)	34 (70.8)	14 (29.2)	
Age (years) Mean ± SD: 34.85 ± 4.74				
19 – 30	29 (17.0)	16 (55.2)	13 (44.8)	0.188
31 – 40	119 (69.6)	82 (68.9)	37 (31.1)	
41 – 50	23 (13.4)	18 (78.3)	5.0 (21.7)	
Marital status				
Single	20 (11.7)	12 (60.0)	8.0 (40.0)	**0.015**
Married	142 (83.0)	102 (71.8)*	40 (28.2)*	
Divorced	8.0 (4.7)	2.0 (25.0)*	6.0 (75.0)*	
Widow	1.0 (0.6)	0.0 (0.0)	1.0 (100)	
Level of education				
Diploma	103 (60.2)	69 (67.0)	34 (33.0)	0.365
Bachelor	60 (35.1)	41 (68.3)	19 (31.7)	
Master	7.0 (4.1)	6.0 (85.7)	1.0 (14.3)	
Doctoral	1.0 (0.6)	0.0 (0.0)	1.0 (100)	
Job title				
Bedside Nurse	123 (71.9)	80 (65.0)	43 (35.0)	0.447
Charge Nurse	39 (22.8)	29 (74.4)	10 (25.6)	
Head Nurse	9.0 (5.3)	7.0 (77.8)	2.0 (22.2)	
Work experience (years)				
Less than 1 year	8.0 (4.7)	7.0 (87.5)	1.0 (12.5)	0.110
1 - 4 years	17 (9.9)	9.0 (52.9)	8.0 (47.1)	
5 - 10 years	56 (32.7)	43 (76.8)	13 (23.2)	
More than 10 years	90 (52.7)	57 (63.3)	33 (36.7)	

Data are expressed as means ± SD for continuous variables and as percentages for categorical variables. The Chi-square test was used to examine differences in the prevalence of different categorical variables. A p-value less than 0.05 was considered statistically significant. SD, standard deviations.

Bold P values means that P value less than 0.05 and it was considered as statistically significant; while the symbol * indicating a difference between these variables.

The significant association between marital status and sex, in bold in **Table 1** (p-value = 0.015) indicates a sex difference for this variable; other associations were not significant (P value > 0.05).

**Table 2** shows that all the nurses in the study (100%) were exposed to workplace violence. Of these, 38.6% (74.2% male vs. 25.8% female) reported being exposed to violence 3 to 10 times. Furthermore, 62.0% experienced verbal violence, while 38.0% encountered physical violence. A significant proportion (47.4%) of the nurses faced violence from patients, 64.3% reported experiencing violence during the morning shift, and 23.4% tried to defend themselves when exposed to violence. Regarding verbal violence, a majority (47.4%) reported being subjected to cursing. As for physical violence, 23.4% of the nurses were hit with hands, and only 0.6% experienced attempted rape or sexual harassment. Additionally, 64.3% of the nurses (75.5% male vs. 24.5% female) reported exposure to violence during the morning shift.

**Table 2:** Prevalence of workplace violence and its characteristics among the study participants by sex (N=171).

Variable	Total, n (%) 171 (100)	Male, n (%) 116 (67.8)	Female, n (%) 55 (32.2)	p-value
Exposure to workplace violence				
Yes	171 (100)	116 (67.8)	55 (32.2)	–
Frequency of occurrence				
1–2 times	77 (45.0)	46 (59.7)	31 (40.3)	0.122
3–10 times	66 (38.6)	49 (74.2)	17 (25.8)	
> 10 times	28 (16.4)	21 (75.0)	7.0 (25.0)	
Type of violence				
Verbal violence	106 (62.0)	74 (69.8)	32 (30.2)	0.294
Physical violence	65 (38.0)	42 (64.6)	23 (35.4)	
Type of verbal violence				
Cursing	81 (47.4)	56 (69.1)	25 (30.9)	0.656
Bullying	9.0 (5.3)	8.0 (88.9)	1.0 (11.1)	
Insults	3.0 (1.8)	2.0 (66.7)	1.0 (33.3)	
Threat	13 (7.5)	8.0 (61.5)	5.0 (38.5)	
No verbal violence	65 (38.0)	42 (64.6)	23 (35.4)	
Type of physical violence				
Hit by hands	40 (23.4)	29 (72.5)	11 (27.5)	0.405
Hold the body and shake it	8.0 (4.7)	4.0 (50.0)	4.0 (50.0)	
Pushing	6.0 (3.5)	4.0 (66.7)	2.0 (33.3)	
Attempted suffocation	3.0 (1.8)	2.0 (66.7)	1.0 (33.3)	
Biting	4.0 (2.3)	1.0 (25.0)	3.0 (75.0)	
Attempted rape or sexual harassment	1.0 (6.0)	0.0 (0.0)	1.0 (100)	
Spitting	3.0 (1.8)	2.0 (66.7)	1.0 (33.3)	
No physical violence	106 (62.0)	74 (69.8)	32 (30.2)	
Person perpetrating the violence (perpetrator)				
Patient	81 (47.4)	57 (70.4)	24 (29.6)	0.224
Patient’s relatives/friends	52 (30.4)	29 (55.8)	23 (44.2)	
Unknown visitor	12 (7.0)	9.0 (75.0)	3.0 (25.0)	
Nurse	11 (6.4)	10 (90.9)	1.0 (9.1)	
Doctor	3.0 (1.8)	2.0 (66.7)	1.0 (33.3)	
Employee	12 (7.0)	9.0 (75.0)	3.0 (25.0)	
Working shift when exposed to violence				
Morning shift	110 (64.3)	83 (75.5)*	27 (24.5)*	**0.017**
Evening shift	47 (27.5)	26 (55.3)	21 (44.7)	
Night shift	14 (8.2)	7.0 (50.0)	7.0 (50.0)	
Response or reaction when exposed to violence				
Did nothing	29 (17.0)	20 (69.0)	9.0 (31.0)	0.202
Told the person to stop	69 (40.4)	42 (60.9)	27 (39.1)	
Tried to defend yourself	40 (23.4)	32 (80.0)	8.0 (20.0)	
Asked for help	19 (11.1)	14 (73.7)	5.0 (26.3)	
Informed the hospital managers	13 (7.6)	8.0 (61.5)	5.0 (38.5)	
Requested leave/transfer	1.0 (0.5)	0.0 (0.0)	1.0 (100)	

Data are expressed as percentages for categorical variables. The Chi-square test was used to examine differences in the prevalence of different categorical variables. A p-value less than 0.05 was considered statistically significant.

Bold P values means that P value less than 0.05 and it was considered as statistically significant; while the symbol * indicating a difference between these variables.

The significant association between sex and working shift during exposure to violence, in bold in **Table 2** (p-value = 0.017), indicates a sex difference; the other variables were not significantly associated with sex (P value > 0.05).

In this study, a 36-item short-form quality of life tool was used to assess the overall quality of life scores. The quality of life scores between the groups (male and female participants) were compared in the study. As shown in **Table 3**, the total mean quality of life score was calculated and compared between male and female nurses, but no statistically significant difference was found between the sexes (P value = 0.349). However, when looking at the specific domain of pain, a statistically significant difference was observed between the male and female nurses (p-value = 0.003), indicating that there was a difference in how pain impacted the quality of life for each group. The other quality of life domains were not significantly associated with sex (P value > 0.05).

**Table 3:** Quality of life scores of the study participants by sex, using the 36-item quality of life tool (short-form) (N=171).

Variables	Total, n (%) 171 (100)	Male, n (%) 116 (67.8)	Female, n (%) 55 (32.2)	p-value
	Mean ± SD	Mean ± SD	Mean ± SD	
1. First section of general health total score	4.01 ± 1.7	4.09 ± 1.8	3.83 ± 1.5	0.369
2. Limitations of activities total score	26.7 ± 4.0	26.5 ± 3.9	27.0 ± 4.3	0.493
3. Physical health total score	6.35 ± 1.4	6.27 ± 1.4	6.52 ± 1.6	0.307
4. Emotional health total score	4.67 ± 1.3	4.56 ± 1.3	4.90 ± 1.2	0.104
5. Social activity total score	4.35 ± 2.2	4.53 ± 2.3	3.98 ± 2.1	0.141
6. Total pain score	4.88 ± 1.4	5.08 ± 1.6*	4.47 ± 1.0*	**0.003**
7. Energy and emotions total score	31.5 ± 7.9	31.9 ± 8.2	30.6 ± 7.2	0.316
8. Second section of general health total score	10.6 ± 2.2	10.7 ± 2.3	10.4 ± 2.0	0.443
**Total quality of life score**	**93.1 ± 11.5**	**93.7 ± 10.9**	**91.9 ± 12.7**	**0.349**

Data are expressed as means ± SD for continuous variables. The differences between means were tested using independent sample t-tests. A p-value less than 0.05 was considered statistically significant. SD, standard deviations.

Bold P values means that P value less than 0.05 and it was considered as statistically significant; while the symbol * indicating a difference between these variables.

**Figure 1:**
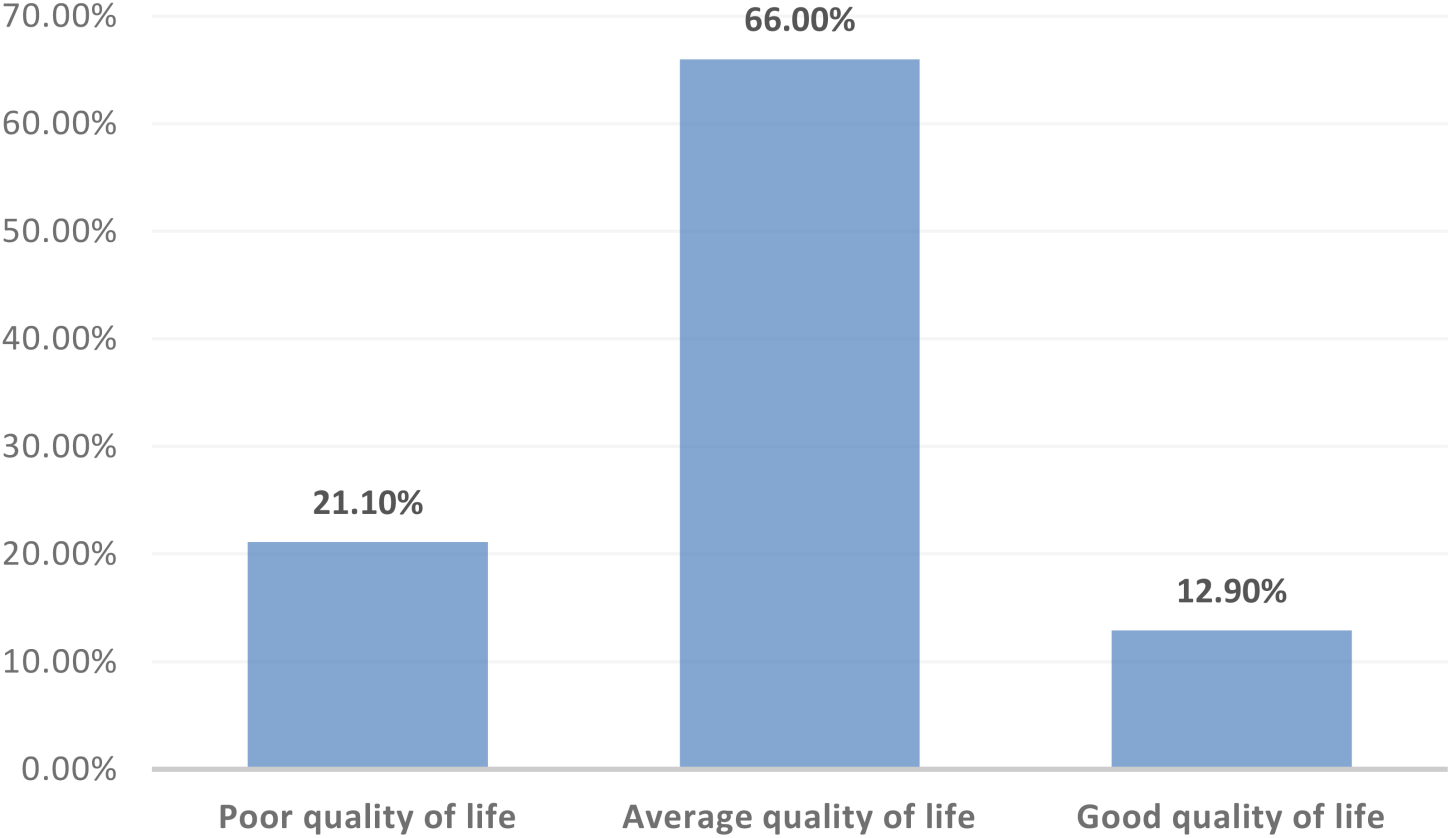
Distribution of the study participants according to their quality of life categories (N=171).

**Figure 1** and **Table 4** show that 66.0% of the nurses in the study reported having an average quality of life, 12.9% had a good quality of life, and 21.1% had a poor quality of life following exposure to workplace violence.

**Table 4:** The association between workplace violence and its characteristics with the quality of life categories (N=171).

Variable	Poor, n (%) 36 (21.1)	Average, n (%) 113 (66.0)	Good, n (%) 22 (12.9)	p-value
Exposure to workplace violence				
Yes	36 (21.1)	113 (66.0)	22 (12.9)	–
Frequency of occurrence				
1–2 times	24 (31.2)	44 (57.1)	9.0 (11.7)	0.050
3–10 times	7.0 (10.6)	49 (74.2)	10 (15.2)	
> 10 times	5.0 (17.9)	20 (71.4)	3.0 (10.7)	
Type of violence				
Verbal violence	30 (28.3)*	58 (54.7)	18 (17.0)*	**0.001**
Physical violence	6.0 (9.2)	55 (84.6)	4.0 (6.2)	
Type of verbal violence				
Cursing	24 (29.6)*	49 (60.5)	8.0 (9.9)*	**0.001**
Bullying	2.0 (22.2)	5.0 (55.6)	2.0 (22.2)	
Insults	2.0 (66.7)*	1.0 (33.3)	0.0 (0.0)*	
Threat	2.0 (15.4)	3.0 (23.1)	8.0 (61.5)	
No verbal violence	6.0 (9.2)	55 (84.6)	4.0 (6.2)	
Type of physical violence				
Hit by hands	3.0 (7.5)	36 (90.0)	1.0 (2.5)	**0.004**
Hold the body and shake it	1.0 (12.5)*	4.0 (50.0)	3.0 (37.5)*	
Pushing	0.0 (0.0)	6.0 (100)	0.0 (0.0)	
Attempted suffocation	0.0 (0.0)	3.0 (100)	0.0 (0.0)	
Biting	0.0 (0.0)	4.0 (100)	0.0 (0.0)	
Attempted rape or sexual harassment	1.0 (100)*	0.0 (0.0)	0.0 (0.0)*	
Spitting	1.0 (33.3)*	2.0 (66.7)	0.0 (0.0)*	
No physical violence	30 (28.3)*	58 (54.7)	18 (17.0)*	
Person perpetrating the violence (perpetrator)				
Patient	17 (21.0)	50 (61.7)	14 (17.3)	0.180
Patient’s relatives/friends	13 (25.0)	37 (71.2)	2.0 (3.8)	
Unknown visitor	2.0 (16.7)	6.0 (50.0)	4.0 (33.3)	
Nurse	1.0 (9.1)	10 (90.9)	0.0 (0.0)	
Doctor	1.0 (33.3)	2.0 (66.7)	0.0 (0.0)	
Employee	2.0 (16.7)	8.0 (66.6)	2.0 (16.7)	
Working shift when exposed to violence				
Morning shift	19 (17.3)	75 (68.2)	16 (14.5)	0.542
Evening shift	13 (27.7)	29 (61.7)	5.0 (10.6)	
Night shift	4.0 (28.6)	9.0 (64.3)	1.0 (7.1)	
The response or reaction when exposed to violence				
Did nothing	7.0 (24.1)	17 (58.6)	5.0 (17.2)	0.464
Told the person to stop	13 (18.8)	47 (68.1)	9.0 (13.0)	
Tried to defend yourself	8.0 (20.0)	27 (67.5)	5.0 (12.5)	
Asked for help	4.0 (21.1)	14 (73.7)	1.0 (5.3)	
Informed the hospital managers	4.0 (30.8)	8.0 (61.5)	1.0 (7.7)	
Requested leave/transfer	0.0 (0.0)	0.0 (0.0)	1.0 (100)	

Data are expressed as percentages for categorical variables. The Chi-square test was used to examine differences in the prevalence of different categorical variables. A p-value less than 0.05 was considered statistically significant. Poor quality of life = < 88.8 total score; average quality of life 88.8 to <111 total score; and good quality of life = ≥111 to 148 total score.

Bold P values means that P value less than 0.05 and it was considered as statistically significant; while the symbol * indicating a difference between these variables.

The significant associations between workplace violence and its characteristics with type of violence, type of verbal violence, and type of physical violence are in bold in **Table 4** (p-values < 0.05 for all). The other characteristics of workplace violence were not significantly associated with the quality of life categories (p-values > 0.05).

## Discussion

To the best of our knowledge, this is one of the first studies to assess the prevalence of verbal and physical workplace violence against psychiatric nurses and its impact on their quality of life in Saudi Arabia. The study found that 100% of the nurses were exposed to workplace violence, with significant associations between the type of violence and the nurses’ quality of life. Specifically, there was a significant correlation between exposure to workplace violence and quality of life, with many nurses reporting average or poor quality of life post-exposure. The high prevalence of workplace violence reported in this study may be attributed to several factors, including the unique challenges faced by psychiatric nurses, cultural and regional variations, differences in study methodology, and the specific characteristics of the study population.

In terms of sociodemographic characteristics, more than two-thirds of the nurses were in the 31–40 age group, with a mean age of 34.85 ± 4.74 years, and the majority were male. This finding aligns with Ding et al. (11), who reported a similar mean age for nurses but contrasts with Sayed et al. (12), where fewer nurses fell into the 31–40 age group. Regarding marital status, the majority of the nurses were married, which is consistent with Ali and Mohamed (13), but differs from Bakr et al. (14), who found a lower percentage of married nurses. Additionally, more than half of the nurses had a nursing diploma, a finding consistent with Kibunja et al. (15) but differing from Weldehawaryat et al. (16), where most nurses held a bachelor’s degree. The study also showed that nearly two-thirds of the nurses were bedside nurses, which contrasts with Sweelam et al. (17), who reported that the majority were staff nurses. The study also revealed that over half of the nurses had more than 10 years of experience, which is in line with Kotti et al. (18), but differed from Al-Kalbani et al. (19), where fewer nurses had over 10 years of experience.

In terms of workplace violence, the study found that 100% of the nurses were exposed to some form of violence, consistent with findings from Mohammed et al. (20) and Bernardes et al. (21). The study also reported that 38.6% of nurses experienced violence 3–10 times, with more than half exposed to verbal violence and less than half to physical violence, primarily from patients. This finding aligns with El-Gamal et al. (22) and Mohammed et al. (20), but contrasts with El‐Hneiti et al. (23), who found lower exposure to violence from patients.

Regarding the timing of violence, the study revealed that two-thirds of nurses were exposed to violence during the morning shift, with only a small percentage attempting to defend themselves. This result is similar to Hassan et al. (21) but differs from Anose et al. (24), where most violence occurred during late shifts. In terms of the type of violence, the study found that nearly half of the nurses experienced verbal violence, primarily cursing, and 23.4% were physically hit by hands.

These results align with Kim et al. (25) and Öztaş et al. (26) but differ from Ose et al. (27), who found threats to be more common. In terms of quality of life, the study revealed that the majority of nurses had an average quality of life, with a significant percentage reporting a poor quality of life after exposure to workplace violence. There was a significant correlation between the type of violence and quality of life, supporting the findings of Galanis et al. (28) and Kim et al. (29), who reported a decline in quality of life among nurses exposed to workplace bullying. Similarly, Itzhaki et al. (4) found a link between workplace violence and reduced quality of life. These findings suggest the need for interventions to improve nurse wellbeing and mitigate the negative effects of workplace violence, with further studies recommended to confirm these results.

Additionally, to reduce violence, particularly from the community, organizations can take several actions. These include implementing community outreach programs to promote mutual respect and raise awareness about the impact of violence on mental health workers. Strengthening communication between healthcare workers and the community can also build trust and reduce misunderstandings. Regular training in de-escalation techniques and improving workplace security with surveillance and security personnel can help prevent violent incidents. Finally, providing a supportive environment with counseling and stress-relief programs ensures that healthcare workers are better prepared to handle challenging situations.

## Conclusion

The study found that a significant portion of psychiatric nurses in Saudi Arabia experienced verbal and physical workplace violence. Moreover, a significant correlation was observed between the type of violence, including both verbal and physical, and the quality of life of the nurses.

In light of these findings, the administration of mental health hospitals should implement strategies to prevent, reduce, and manage workplace violence, and address its adverse effects on the nurses’ quality of life. Nurses should receive training on handling violent situations, focusing on communication, de-escalation, and managing aggression. This will also help them understand their rights and reporting mechanisms. Moreover, clear, accessible procedures for reporting workplace violence should be established, ensuring nurses feel supported and protected when incidents are reported.

### Strengths and limitations

The primary strength of our study lies in its status as one of the first to examine the prevalence of verbal and physical workplace violence against psychiatric nurses and its impact on their quality of life in Saudi Arabia. However, like most cross-sectional studies, a limitation of our research is that it cannot establish causal relationships. Unfortunately, we did not identify and control for potential confounding variables that may have influenced the results.

## Data Availability

The raw data supporting the conclusions of this article will be made available by the authors, without undue reservation.
