# Supplementary material for: The prevalence of workplace violence toward psychiatric nurses in Saudi Arabia and its effect on their quality of life
Source: Front Psychiatry. 2025 Mar 20;16:1524845. doi: 10.3389/fpsyt.2025.1524845 (PMC11965361; doi:10.3389/fpsyt.2025.1524845)
Supplement: Supplementary file 1 [file DataSheet1.pdf]

QUESTIONNAIRE

Hospital name: ………………………………………..

Part I: Demographic Soci-Economic Characteristics of The Study Participants

1- Age (years):

19 – 30 ☐ 31-40 ☐

41-50 ☐ ≥51 ☐

2- Gender: **-** Male ☐ **-** Female ☐

3- Marital status: - Single ☐ - Married ☐

- Divorced ☐ - Widow ☐

4- Level of education:

- diploma ☐ - Bachelor of nursing ☐

- Master degree ☐ - Doctoral degree ☐

5- Job title: - Bedside nurse ☐ - Charge nurse ☐

- Head nurse ☐

6- years of experience: - < 1year ☐ - 1- 4 years ☐

- 5-10 years ☐ - >10 years ☐

Part II: Prevalence of work place violence and its characteristics among nurses:

1- Exposure to violence:

- Yes ☐ - No ☐

2- Frequency of occurrence:

- 1-2 times ☐ - 3-10 times ☐ - >10 times ☐

3- Type of violence:

- Verbal violence ☐
- Physical violence ☐

4- Perpetrators:

- Patient ☐
- Patient relatives/friends ☐
- Nurse ☐
- Doctor ☐
- Employee ☐
- Unknown visitors ☐

5- Work shift when you exposure to violence

- Morning shift ☐
- Evening shift ☐

- Night shift ☐

6- Reaction to the violence act

- Did nothing ☐
- Told the person to stop ☐
- Tried to defend oneself ☐
- Asked for help ☐
- Reported to hospital administration ☐
- Request for vacation/transfer ☐

7- Verbal and physical violence

Items	Yes	No
**Verbal violence**		
1. Cursing		
2. Bullying		
3. Insults		
4. Threat		
**Physical violence**		
1. Hit		
2. Shaken		
3. Struck with an object		
4. Kicked		
5. Pushed		
6. Attempted strangling		
7. Bitten		
8. Attempted rape		
9. Stabbed with knife		

10.Spat on		
11.Others remember…………………….		

8- Response after exposure to a WPV incident

Items	Yes	No
1. No response		
2. Talking to family and friends		
3. Request to be transferred to another department		
4. Pretending nothing happened		
5. Getting professional help		
6. Getting help from the police		
7. Warning the patient		
8. Talking with colleagues		
9. Reporting incident to the manager		
10.Reacting with violence		
11.Getting legal help		

The medical outcomes study 36-item quality of life health survey short-form

For each of the following questions, please circle the number that best describes your answer.

1- First section of General health

**1. In general, would you say your health is:**
Excellent
Very good
Good
Fair
Poor
**2. Compared to one year ago, how do you rate your health in general now that you have been exposed to violence at work?**
Much better now than one year ago
Somewhat better now than one year ago
About the same

Somewhat worse now than one year ago	
Much worse now than one year ago	

2- Limitations of activities

The following items relate to activities you might do on a typical day. Do you see that your health now restricts you in practicing these activities as a result of being affected by violence at work? If so, to what extent does it affect?

	**Yes, Limiteda Lot**	**Yes, Limiteda Little**	**No, not limited at All**
a. **Vigorous activities**, such as running, lifting heavy objects, participating in strenuous sports			
b. **Moderate activities**, such as moving a table, pushing a vacuum cleaner, bowling, or playinggolf			
c. Lifting or carrying groceries			
d. Climbing **several** flights of stairs			
e. Climbing **one** flight of stairs			
f. Bending, kneeling, or stooping			
g. Walking **more than a mile**			
h. Walking **several blocks**			
i. Walking **one block**			
j. Bathing or dressing yourself			

3- Physical health problems

During the **past 4 weeks**, have you had any of the following problems with your work or other regular daily activities **as a result of violence exposure at work**?

	**Yes**	**No**
a. Cut down the amount of time you spent on work or other activities		

b. **Accomplished less** than you would like		
c. Were limited in the **kind** of work or other activities		
d. Had **difficulty** performing the work or other activities (for example, it took extra effort)		

4- Emotional health problems

During the past 4 weeks, have you had any of the following problems with your work or other regular daily activities as a result of any emotional problems as a result of exposure to violence in work place (such as feeling depressed or anxious)?

	**Yes**	**No**
a. Cut down the amount of time you spent on work or other activities		
b. **Accomplished less** than you would like		
c. Didn't do work or other activities as **carefully** as usual		

5- Social activities

1- During the past four weeks, to what extent did your health condition, physically and psychologically due to exposure to violence in work place, affect your normal social relationships with family, friends, neighbors and groups?

- Not at all
- Slightly
- Moderately
- Quite a bit
- Extremely

2- During the past four weeks, how did your health and psychological condition as a result of violence exposure in work place affect your practice of social activities such as visiting friends and family?

- Not at all

- Slightly
- Moderately
- Quite a bit
- Extremely

6- Pain

Items
**How much bodily pain have you had as a result of violence in work place during the past 4 weeks?**
None
Mild
Moderate
Severe
Very severe
**During the past 4 weeks, how much did pain as a result of exposure to pain in work place interfere with your normal work (including both work outside the home and housework)?**
Not at all
A little bit
Moderately
Quite a bit
Extremely

7- Energy and emotions

How much of the time during the past 4 weeks . . .

	All of the Time	Most of the Time	A Good Bit of the Time	Some of the Time	A Little of the Time	None of the Time
a. Did you feel full of pep?						
b. Have you been a very nervous person?						

c. Have you felt so down in the dumps that nothing could cheer you up?						
d. Have you felt calm and peaceful?						
e. Did you have a lot of energy?						
f. Have you felt downhearted and blue?						
g. Did you feel worn out?						
h. Have you been a happy person?						
i. Did you feel tired?						

8- Second section of general health

How true or false is each of the following statements for you?

	Definitely True	Mostly True	Don't Know	Mostly False	Definitely False
a. I seem to get sick a little easier than other people					
b. I am as healthy as anybody I know					
c. I expect my health to get worse					
d. My health is excellent					

Variables	Total, n (%)	Males, n (%)	Females, n (%)	P value

	171 (100)	116 (67.8)	55 (32.2)	
**Section I: General health**				
**In general, would you say your health is?**				
Excellent				
Very good				
Good				
Fair				
Poor				
**Compared to one year ago, how do you rate your health in general now that you have been exposed to violence at work?**				
Much better now than one year ago				
Somewhat better now than one year ago				
About the same				
Somewhat worse now than one year ago				
Much worse now than one year ago				
**Section II: Limitations of activities; Do you see that your health now restricts you in practicing these activities as a result of being affected by violence at work? If so, to what extent does it affect?**				
**Vigorous activities, such as running, lifting heavy objects, participating in strenuous sports**				
Yes, limited a lot				
Yes, limited a little				
No, not limited at all				
**Moderate activities, such as moving a table, pushing a vacuum cleaner, bowling, or playing golf**				
Yes, limited a lot				
Yes, limited a little				
No, not limited at all				
**Lifting or carrying groceries**				
Yes, limited a lot				
Yes, limited a little				
No, not limited at all				
**Climbing several flights of stairs**				
Yes, limited a lot				
Yes, limited a little				
No, not limited at all				

Thank You So Much For Your Sincere Cooperation
